# CHATO National Trial: Using Leadership Closeout Meetings to Improve Online Education in Nursing Homes

**DOI:** 10.1093/geroni/igaf122.157

**Published:** 2025-12-31

**Authors:** Carissa Coleman, Maria Jaros, Maria Roche-Dean, Amy Berkley, Frances Yang, Clarissa Shaw, Kristine Williams, Maria Hein

**Affiliations:** University of Kansas Medical Center, Kansas City, Kansas, United States; University of Kansas Medical Center, Kansas City, Kansas, United States; Kirkhof College of Nursing, Grand rapids, Michigan, United States; University of Kansas School of Nursing, Kansas City, Kansas, United States; University of Kansas, University of Kansas Medical Center, Kansas, United States; University of Iowa College of Nursing, Iowa City, Iowa, United States; University of Kansas, Kansas City, Kansas, United States; University of Iowa, Iowa City, Iowa, United States

## Abstract

The Changing Talk: Online (CHATO) intervention was developed to increase staff access to person-centered dementia care communication education in a national nursing home sample. A qualitative review of meeting notes taken from the closeout meetings (N = 75) with nursing home implementation leads was performed to determine: 1) acceptability of the modules, and 2) facilitators and barriers associated implementation, to improve uptake across nursing homes. The open-ended interviews completed over the 3 year study provided clinical leaders the opportunity to give feedback on the educational content, technical aspects of the modules, implementation strategies, and what leaders liked most and least about the program. CHATO training facilitators identified: (93.3%) of implementation leads reported the content was useful, followed by the videos illustrating behavioral examples (12.0%), and the training was useful for onboarding staff (9.3%). CHATO training barriers identified: training was too long (48.0%), login issues (30.7%), navigation (29.3%), “good refresher”(18.7%), not cell phone compatible (17.3%), and low computer literacy (16.0%). Implementation facilitators identified were site specific, tailored strategies (32.0%), rewards (22.7%), onsite discussions (17.3%), mandatory training approach (14.7%), and weekly reminders (12.0%). Barriers to implementation were lack of interest (18.7%), training was too time consuming (14.7%), too many other online trainings (10.7%), and staff were too busy (10.7%). These findings will provide input for the redesign of the CHATO modules and implementation plan for the next iteration of the Changing Talk education intervention.

